# Supplementary figures and images for: Quantifying male and female pheromone-based mate choice in Caenorhabditis nematodes using a novel microfluidic technique
Source: PLoS One. 2017 Dec 13;12(12):e0189679. doi: 10.1371/journal.pone.0189679 (PMC5728554; doi:10.1371/journal.pone.0189679)

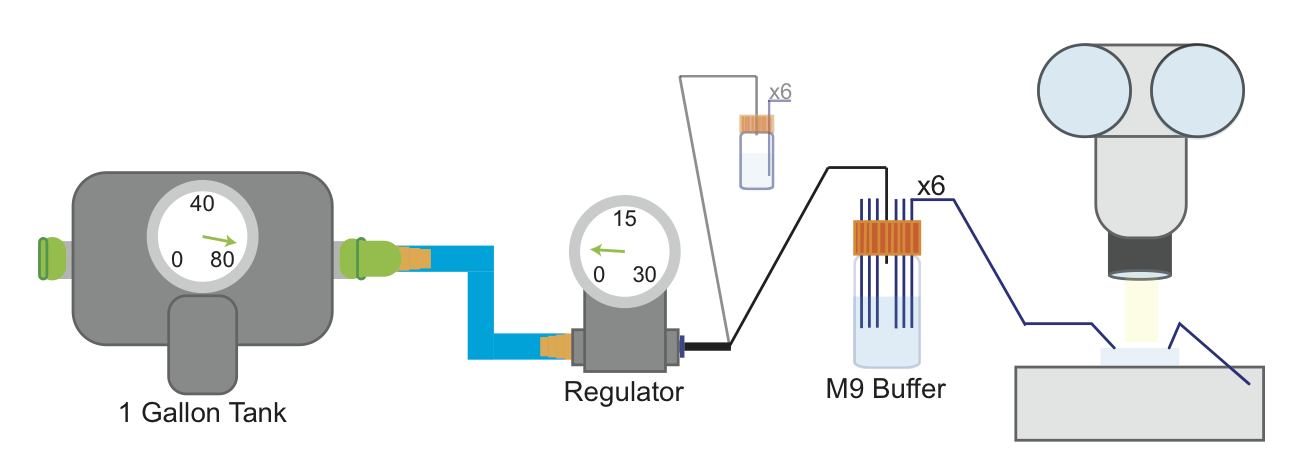

Supplement: S1 Fig — A pressurized air system was used to maintain a constant flow rate through the microfluidic devices. A one gallon air tank was regulated to 1.5 PSI was sufficient to run experiments for up to 3 hours. The air-line running from the tank was bifurcated to pressurize two sealed 500mL bottles of M9 buffer. The bottle caps were modified to supply six liquid lines as well as hold the air-line, which terminated at the cap. Since the Pheromone Arena requires three liquid inlet lines, each M9 bottle could run two devices and therefore a single one gallon air tank could simultaneously run four devices. The tubing lengths were equal for each partition of the set-up to maintain equal flow through all lines. The Pheromone Arena was kept on a confocal microscope at 20°C for the duration of each experiment. This figure was modified with permission from Stephen Banse. (TIFF) [file pone.0189679.s003.tiff]

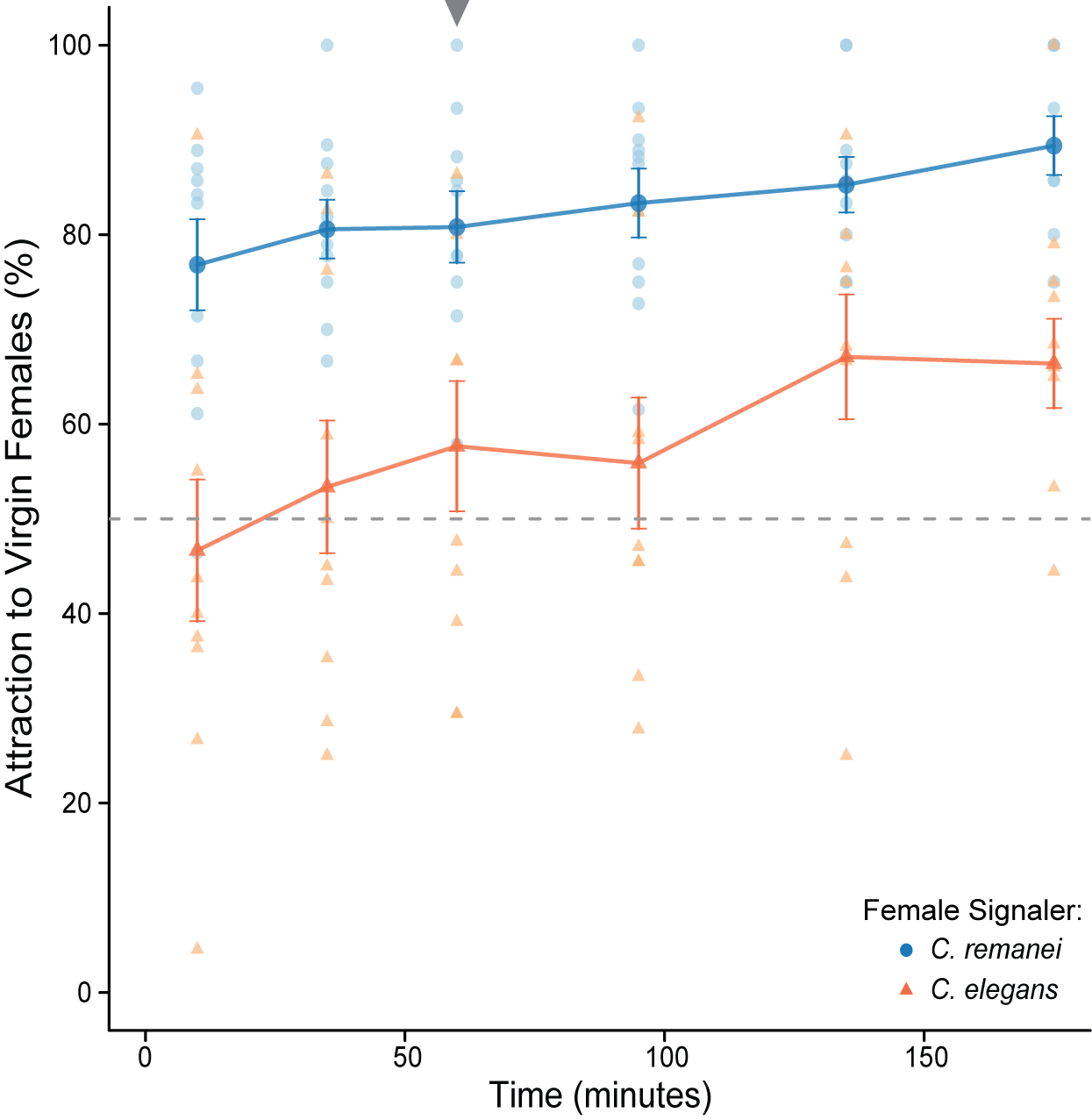

Supplement: S2 Fig — Virgin, day 2 adult C. remanei males were given a choice between virgin and mated conspecific female pheromone (blue) and C. elegans males were given a choice between virgin and mated conspecific female pheromone (orange). The weighted means and standard error are plotted over time. By 60 minutes into the experiment males had made a consistent choice (down triangle). The null hypothesis of no choice is given by the dashed line. In each assay males were more attracted to virgin females than mated ones. (TIF) [file pone.0189679.s004.tif]

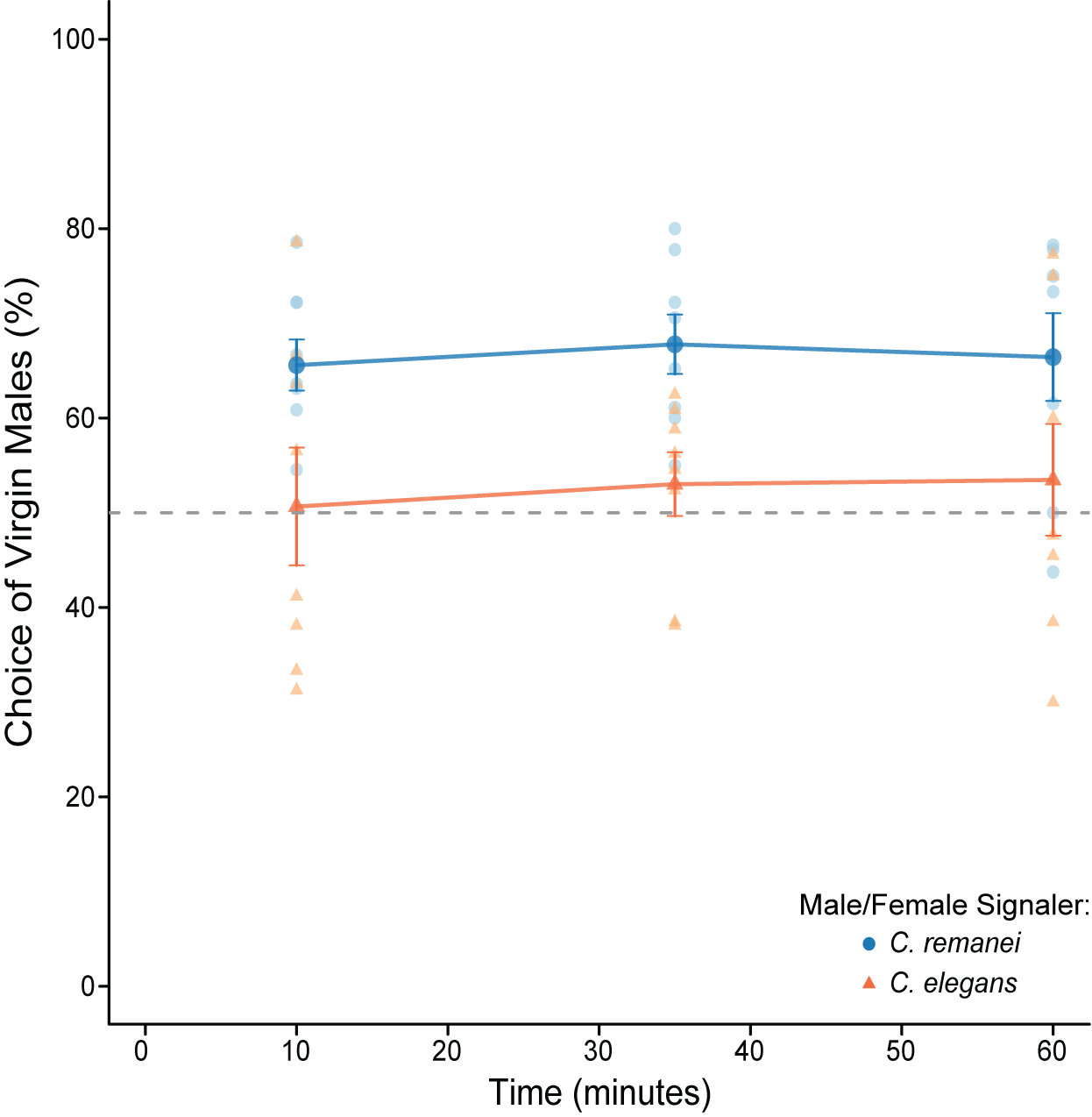

Supplement: S3 Fig — Virgin, day 1 adult C. remanei females were given a choice between virgin conspecific male and female pheromones (blue) and C. elegans males were given a choice between virgin conspecific male and female pheromones (orange). The weighted means and standard error are plotted over time. The null hypothesis of no choice is given by the dashed line. Only C. remanei females made a measurable choice of male pheromone over female pheromone. (TIF) [file pone.0189679.s005.tif]
